# Sustainable Survival for adolescents living with HIV: do SDG‐aligned provisions reduce potential mortality risk?

**DOI:** 10.1002/jia2.25056

**Published:** 2018-02-27

**Authors:** Lucie Cluver, Marija Pantelic, Mark Orkin, Elona Toska, Sally Medley, Lorraine Sherr

**Affiliations:** ^1^ Department of Social Policy and Intervention University of Oxford Oxford United Kingdom; ^2^ International HIV/AIDS Alliance Hove United Kingdom; ^3^ Development Pathways to Health Research Unit University of Witwatersrand Johannesburg South Africa; ^4^ AIDS and Society Research Unit University of Cape Town Cape Town South Africa; ^5^ Department of Global Health University College London London United Kingdom

**Keywords:** HIV/AIDS, SDGs, adolescents, viral load, tuberculosis, South Africa, social protection

## Abstract

**Introduction:**

The Sustainable Development Goals (SDGs) present a groundbreaking global development agenda to protect the most vulnerable. Adolescents living with HIV in Sub‐Saharan Africa continue to experience extreme health vulnerabilities, but we know little about the impacts of SDG‐aligned provisions on their health. This study tests associations of provisions aligned with five SDGs with potential mortality risks.

**Methods:**

Clinical and interview data were gathered from N = 1060 adolescents living with HIV in rural and urban South Africa in 2014 to 2015. All ART‐initiated adolescents from 53 government health facilities were identified, and traced in their communities to include those defaulting and lost‐to‐follow‐up. Potential mortality risk was assessed as either: viral suppression failure (1000+ copies/ml) using patient file records, or adolescent self‐report of diagnosed but untreated tuberculosis or symptomatic pulmonary tuberculosis. SDG‐aligned provisions were measured through adolescent interviews. Provisions aligned with SDGs 1&2 (no poverty and zero hunger) were operationalized as access to basic necessities, social protection and food security; An SDG 3‐aligned provision (*ensure healthy lives*) was having a healthy primary caregiver; An SDG 8‐aligned provision (*employment for all*) was employment of a household member; An SDG 16‐aligned provision (*protection from violence*) was protection from physical, sexual or emotional abuse. Research partners included the South African national government, UNICEF and Pediatric and Adolescent Treatment for Africa.

**Results:**

20.8% of adolescents living with HIV had potential mortality risk – i.e. viral suppression failure, symptomatic untreated TB, or both. All SDG‐aligned provisions were significantly associated with reduced potential mortality risk: SDG 1&2 (OR 0.599 CI 0.361 to 0.994); SDG 3 (OR 0.577 CI 0.411 to 0.808); SDG 8 (OR 0.602 CI 0.440 to 0.823) and SDG 16 (OR 0.686 CI 0.505 to 0.933). Access to multiple SDG‐aligned provisions showed a strongly graded reduction in potential mortality risk: Among adolescents living with HIV, potential mortality risk was 38.5% with access to no SDG‐aligned provisions, and 9.3% with access to all four.

**Conclusions:**

SDG‐aligned provisions across a range of SDGs were associated with reduced potential mortality risk among adolescents living with HIV. Access to multiple provisions has the potential to substantially improve survival, suggesting the value of connecting and combining SDGs in our response to paediatric and adolescent HIV.

## 1 Introduction

The Sustainable Development Goals (SDGs) signal a radical change for the paediatric and adolescent HIV sector. We have moved from an HIV‐focused Millennium Development Goal to a new agenda that incorporates wider health needs within a multifaceted set of human capital targets ‐ ranging from education to gender equality to violence prevention. Importantly, the SDGs also prioritize integration and partnership between the goals and their targets 1.

In sub‐Saharan Africa, where 60% of adolescents living with HIV reside 2, survival remains a major concern. Adolescent rates of adherence and retention in care remain low 2, 3, 4, 5, and viral suppression failure 6 and TB are common 7. AIDS is the leading cause of death among adolescents in Africa, and adolescent AIDS‐related mortality rates are not decreasing 8. Systematic reviews show few effective interventions for adolescents living with HIV 9, 10, and a primary focus on healthcare provision 11. But qualitative data suggests that adolescents living with HIV do not see health as their only concern, and instead regard themselves as multifaceted individuals with holistic needs 12. In line with this, new WHO guidelines see adolescent health outcomes as a result of intertwined experiences in multiple spheres of their lives 13. Could then a shift to the wider‐ranging focus of the SDGs benefit adolescent HIV care and potential for long‐term survival? And does integration across goals and targets bring risks or opportunities for this exceptionally vulnerable group?

We do not yet have data on adolescents living with HIV that is specifically designed to assess impacts of attainment of SDG targets. However, an existing large‐scale community study of adolescents living with HIV in South Africa 14, 15 provides an opportunity to examine existing services that directly correlate to the contribution of achieving SDG targets, and whether they are associated with reductions in potential mortality risks. Of course, it will be important that future studies specifically measure whether SDG targets are met among adolescents living with HIV at national and international levels, but this study aims to provide valuable input as the world moves towards operationalization of the SDGs. We use adolescents living with HIV as the unit of analysis, examining whether and how individual‐level access to SDG‐aligned provisions affects potential mortality risk (operationalized as viral failure or symptomatic untreated TB).

## 2 Methods

The study was designed in partnership with the South African National Departments of Health, Social Development and Basic Education and National AIDS Council, UNICEF, PEPFAR‐USAID, and Pediatric Adolescent Treatment for Africa (PATA). We conducted a total population sampling survey of adolescents living with HIV in a health district of South Africa's Eastern Cape, a province characterized by poor infrastructure and limited service access. The study included ART‐initiated adolescents, irrespective of whether they were engaged in care at the time of the survey. In order to do this we targeted one urban/peri‐urban/rural health district and visited all health facilities providing HIV services (hospitals, community health centres, and primary care clinics). Of these, all 53 health facilities that provided ART for adolescents were sampled. In each health facility, we went through paper and computerized records to identify all adolescents aged 10 to 19 that had ever initiated ART.

From March 2014 to September 2015, adolescents were traced to their communities and invited to participate in a study of young people, health and social services in South Africa. Of 1202 eligible adolescents, 90.1% (n = 1060) were interviewed. 3.7% of adolescents were untraceable, primarily due to inaccurate names and addresses in clinic files. 0.9% were unable to be interviewed due to very severe developmental disability, and 4.1% refused to participate (either adolescent or primary caregiver), 1.2% were excluded for other reasons. In order to prevent stigma, HIV was not mentioned in recruitment, and neighbouring adolescents were also interviewed (n = 467, not included in analyses). Community‐based tracing resulted in a sample that included high proportions of ART‐initiated adolescents who do not regularly attend facilities, default from treatment, or missed recent appointments (44% of the sample).

Ethical approval was given by the Universities of Cape Town (CSSR 2013/4) and Oxford (SSD/CUREC2/12‐21), the Provincial Departments of Health and Education and ethical review committees of participating facilities. Full voluntary informed consent was obtained from both adolescents and their primary caregivers, and included interviews and access to clinical records. Given low levels of literacy, consent procedures were additionally read aloud. The study did not use financial incentives for participation, but all adolescents received a small gift pack, refreshments and a certificate of participation. Participants were informed that all responses were confidential except in the case of risk of harm to the adolescent or someone else. Where participants or caregivers reported abuse, recent attempted suicide, active untreated TB or other serious risks, immediate referrals and follow‐up were made to health, police and social services, including taking the participant to a health facility when health or abuse‐related cases were reported. 69 high‐risk referrals were made. These included 38 for severe food insecurity, 22 for psychosocial or family issues, 6 for sexual abuse, 5 for suicide attempts, 5 for physical abuse, 4 for extreme illness and 2 for drug use.

Clinical records identified most recent viral load measures, diagnoses and treatment of tuberculosis. With the support of interviewers, adolescents participated in a tablet‐based questionnaire lasting approximately 90 minutes. Standardized measures were translated into Xhosa, back‐translated and provided in both Xhosa and English according to participant choice. The questionnaire was designed in collaboration with our Teen Advisory Group of 20 adolescents to be engaging and adolescent‐friendly, and was pre‐piloted with a further 25 adolescents living with HIV in the Eastern Cape. Interviewers were trained in working with HIV‐affected adolescents and their families.

### 2.1 Measures

*Potential mortality risk* for adolescents living with HIV was assessed using two key predictors: treatment failure and symptomatic pulmonary tuberculosis 16, 17. Antiretroviral treatment failure was operationalized as virologic suppression failure in the past two years (defined as viral load 1000 +/ml 18). Viral load data were extracted from clinic records, however limited health service capacity in the Eastern Cape meant that viral load tests were not consistently performed or recorded: only 673 adolescents (64.5% of the sample) had any viral load recorded in their patient files. Furthermore, only 412 adolescents (38.6%) had a viral load test in the past two years. Viral load measurements that had been taken more than two years prior to the study were excluded. In the very few cases where adolescents had more than one viral load taken in the past two years, the most recent viral load was selected.

Symptomatic pulmonary TB – the leading cause of death among HIV‐infected populations in the region – was unable to be assessed through clinical records as almost none reported any TB testing or results. Consequently, TB was measured as 1) adolescent‐reported TB diagnosis without subsequent treatment or 2) self‐reported current symptoms of TB using WHO diagnostic criteria, validated among 8979 participants in Zimbabwe against two sputum specimens 19, 20. This study identified that positive predictive value (PPV) was highest for the symptom combination of chronic cough and weight loss (sensitivity 72.9%, specificity 85%, PPV 11.4). Negative predictive value (NPV) was highest for the symptom combination of any cough, drenching night sweats, and weight loss (sensitivity 75%, specificity 82.4%, NPV 99.2). Area under ROC curves was estimated to provide a summary measure of diagnostic accuracy, with AUC of 0.81 for HIV‐positive TB. We required fulfilment of criteria for both positive and negative predictive values to maximize precision for each case identification of TB.

We identified provisions aligned with SDG targets that may have potential to improve adolescent outcomes, using systematic reviews and studies of risk and protective factors for child, adolescent and adult HIV‐outcomes 21, 22, 23. First, adult studies suggest that poverty may present a barrier to HIV healthcare access through lack of transport and food 24, 25, 26 and that social protection provision may have potential to improve retention in care 27, 28. Given the overlap between ending poverty, food insecurity and social protection, we combined provisions aligned to SDGs 1 and 2 (“End poverty” and “end hunger”). These were operationalized as access to all of the following: eight basic necessities for children as endorsed by over 80% of the population in the nationally representative SA Social Attitudes Survey, including “3 meals per day” and “free school;” and access to a child‐focused grant (child support or foster child grant) in the household. These provisions align to SDG targets 1.1 (“end extreme poverty”), 1.2 (“reduce […] poverty in all its dimensions”), 1.3 (“Implement nationally appropriate social protection”) and 2.1 (“end hunger”).

Second, studies of HIV‐affected families find improved outcomes for children with healthy and surviving caregivers 29, 30. Consequently, we identified a surviving and healthy caregiver as a provision aligned with SDG 3 (“Ensure healthy lives”). This was measured through adolescent self‐report of having a surviving parent or caregiver taking care of them at home, who was not suffering from chronic illness. This provision is aligned with SDG targets 3.1 (“reduce maternal mortality”) and 3.2 (“end the epidemics of AIDS, tuberculosis, malaria and neglected tropical diseases”). Adolescent health was not included as a potential provision due to high risk of confounding with the study outcomes of viral failure and TB.

Third, studies suggest that caregiver capacity to provide sustained household income can lead to improved child outcomes in the context of HIV 31. Consequently, we included the potential provision of household employment, as aligned with SDG 8 (“Employment… for all”). This was operationalized as adolescent report of at least one employed person in their household.

Fourth, there is now substantial evidence demonstrating that violence has negative health impacts 32, and that protection from abuse may improve adolescents’ psychological and physical capacity to engage with healthcare. Thus, we included a provision aligned with SDG 16 (“Peace and justice,” specifically “end abuse… and all forms of violence against… children”). Never having been physically, emotionally or sexually abused was aligned with targets 16.1 (“significantly reduce all forms of violence”) and 16.2 (“end abuse, exploitation, trafficking and all forms of violence against…children”), and measured through adolescent self‐report using the UNICEF Measures for National‐level Monitoring of Orphans and Other Vulnerable Children and the Juvenile Victimization Questionnaire (JVQ), both used previously in South Africa 33.

#### 2.1.1 Covariates

*Age* (10 to 14 versus 15 to 19), *gender, and urban/rural location* were measured using items based on South Africa's Census 34. *Mode of HIV‐infection* was measured using clinical algorithms, with adolescents coded as vertically infected if they had initiated ART prior to age 12 or if they had been on ART for more than 5 years, based on the year of widely available ART access in the study area 35, 36. *Time on ART* was measured via self‐report. Based on evidence of higher defaulting risk in the first year of treatment 37, a dummy variable was computed to differentiate between 1: “more than one year on treatment” and 0: “one year or less.”

### 2.2 Analysis

Analyses were conducted in six stages in SPSS 22 and STATA 14. First, eligible participants included in the study (90.1%) were compared to those excluded (not found or refused participation) on known socio‐demographic characteristics (age, gender and urban/rural household location) using χ^2^ tests. Second, frequency distributions for high mortality risk among adolescents living with HIV, each of the hypothesized SDG‐aligned provisions, socio‐demographic and HIV‐related covariates were reported. Third, to test initial associations of each SDG‐aligned provision against potential mortality risk among adolescents living with HIV, bivariate logistic regressions were run. Fourth, a multivariate logistic regression was run, including all SDG‐aligned provisions simultaneously and controlling for all potential covariates. All potential two‐way and three‐way interactions were tested in logistic regressions.

Fifth, to compute combined effects of multiple SDG‐aligned provisions the following steps were taken, using provisions found to be significantly associated with reduced mortality risk in Stage 3. First, categorical principal components analysis established that all provisions loaded onto a first component (eigenvalue 1.2, 24.4% of total variance). Loadings for each provision were >0.35. To assess combined effects of multiple SDG‐aligned provisions, a summative index was computed, weighted by the respective component loadings. This weighted summative index was highly correlated with a simple summation of the unweighted dichotomies (Spearman's *rho*, 0.961; *p *<* *0.001), and therefore, for ease of interpretation, the unweighted scale was used. Sixth, a marginal effects model was run to assess predicted probabilities of high mortality risk by combined effects of multiple SDG‐aligned provisions, holding all socio‐demographic and HIV‐related co‐factors at mean values. This was plotted with 95% confidence intervals.

## 3 Results

No differences were found between included and excluded participants on age, gender or rural/urban location (Table 1). Table 2 shows socio‐demographic and HIV‐related characteristics, potential mortality risk and access to SDG‐aligned provisions. Adolescents had a mean age of 13.8 (SD 2.8), were 55% female and 67% vertically infected. 70.9% had been on treatment for >1 year. 20.8% of adolescents had potential mortality risk – either viral failure (8.8% of the full sample), or symptomatic untreated tuberculosis (13.7%). Only 38.6% of the adolescents had results of a viral load test recorded in their clinic files within the past two years. Access to SDG‐aligned provisions varied: 15.2% accessed basic necessities, food security and social protection (SDGs 1 and 2), 76.4% had a healthy and surviving caregiver (SDG 3), 66.6% had someone working within their household (SDG 8) and 52% reported no exposure to physical, emotional or sexual abuse (SDG 16).

**Table 1:** Comparisons between reached and unreached adolescents

	HIV+ (n = 1060)	Excluded (n = 116)	Comparison tests^a^
Age (mean, SD)	13.8, 2.834	14.8, 2.91	*p* = 0.671
Female (n, %)	587, 55.2%	66, 56.9%	*p* = 0.769
Rural (n, %)	228, 21.4%	26, 22.4%	*p* = 0.813

a *p* values associated with z score and χ^2^ tests.

**Table 2:** Socio‐demographic covariates, mortality risk and access to SDG‐aligned provisions

	n, %
Under 15	659, 62.2
Female	584, 55.1
Rural household location	228, 21.5
> 1 year on treatment	753, 70.9
Vertically infected	708, 66.8
Potential mortality risk	221, 20.8
Viral load failure	93, 8.8
TB	145, 13.7
SDG‐aligned provisions	
SDG1 + 2 (basic necessities, food security and social protection)	161, 15.2
SDG3 (caregiver alive and healthy)	810, 76.4
SDG8 (household access to work)	706, 66.6
SDG16 (no child abuse victimization)	551, 52.0
Combined effects of multiple SDG‐aligned provisions	
No SDG‐aligned provisions	53, 5.0
One SDG‐aligned provision	217, 20.5
Two SDG‐aligned provisions	425, 40.1
Three SDG‐aligned provisions	299, 28.2
All four SDG‐aligned provisions	66, 6.2

Bivariate associations between SDG‐aligned provisions and potential mortality risk are presented in Table 3. Multivariate logistic regression (Table 4) showed that all SDG‐aligned provisions remained significantly associated with lower mortality risk for adolescents living with HIV. This was independent of each of the other SDG‐aligned provisions, as well as all socio‐demographic and HIV‐related covariates. Effect sizes were similar across SDGs, ranging from the highest effect OR = 0.57 (SDG 3) to the lowest OR = 0.68 (SDG 16). No covariates were significant in the multivariate model. The Hosmer‐Lemeshow Test indicated good model fit (χ^2^ (df) = 10.425 8, *p = *0.236). No interaction terms showed significant effects.

**Table 3:** Model 1: Bivariate regressions between SDG‐aligned provisions and potential mortality risk

SDG‐aligned provisions	Unadjusted OR	Lower CI	Upper CI
SDG 1 + 2 basic necessities & social protection	0.546**	0.367	0.814
SDG 3 healthy caregiver	0.510***	0.380	0.683
SDG 8 household access to work	0.655**	0.500	0.857
SDG 16 no emotional, physical or sexual abuse	0.650***	0.501	0.843

**Indicates significance at *p* < 0.005; ***indicates significance at *p* < 0.001.

**Table 4:** Model 2: Multivariate regression predicting mortality risk with all hypothesized covariates and SDG‐aligned provisions included

	AOR	Lower CI	Upper CI
Socio‐demographic covariates			
Younger than 15	0.742	0.523	1.05
Female	0.750	0.548	1.02
Rural location	1.28	0.896	1.83
HIV‐related covariates			
Vertical HIV infection	1.25	0.749	2.10
> 1 year on treatment	0.875	0.533	1.43
SDG‐aligned provisions			
SDG 1 + 2 basic necessities & social protection	0.599*	0.361	0.994
SDG 3 healthy caregiver	0.577***	0.411	0.808
SDG 8 household access to work	0.602***	0.440	0.823
SDG 16 no emotional, physical or sexual abuse	0.686**	0.505	0.933

*Indicates significance at *p* < 0.05; **indicates significance at *p* < 0.005; ***indicates significance at *p* < 0.001.

**Figure 1:**
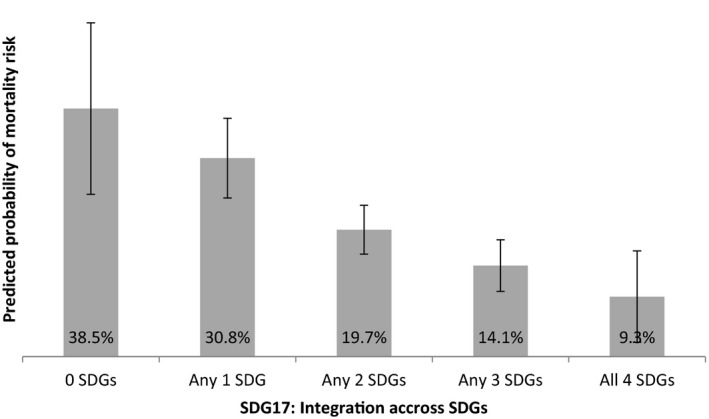
Predicted probabilities of adolescent potential mortality risk by combined SDG‐aligned provisions, controlling for socio‐demographic and HIV‐related co‐factors (n = 1060).

Potential combined effects of access to multiple provisions were tested in a marginal effects model (Figure 1). Independent of all socio‐demographic and HIV‐related covariates, access to a greater number of SDG‐aligned provisions was associated with reduced mortality risk among adolescents living with HIV. There was a clearly graded relationship: with access to none of the provisions, 38.5% of adolescents living with HIV were at potential mortality risk. With access to all four SDG‐aligned provisions, 9.3% of adolescents living with HIV were at potential mortality risk.

## 4 Discussion

This study asks: does an agenda triggered by the SDGs hold out hope for adolescents living with HIV in Southern Africa? First, findings show strong associations of reduced mortality risk with provisions aligned to socio‐economic, family and violence prevention SDGs. These suggest that, whilst treatment access and health system responses are essential, the broader vision of the SDGs may support the long‐term survival of adolescents living with HIV. Second, findings show high rates of severe health deficiency among a large sample of ART‐initiated adolescents recruited from over 50 health facilities in South Africa. With substantial global populations of adolescents living with HIV 38 and a youth explosion within Africa reaching 435 million adolescents by 2050 39, addressing adolescent HIV will be essential if we are to protect the most vulnerable in the region. Third, findings show that access to key SDG‐aligned provisions – social protection, caregiver health, household employment and protection from violence – has the potential to improve critical aspects of health among adolescents living with HIV. The SDG agenda provides a pathway of hope and provision for this group and this study indicates that some of the interventions are to hand and can be incorporated into services to good effect. Moreover, exposure to multiple provisions is associated with greater health benefits for adolescents living with HIV. This suggests potential added value to policymakers, via synergies between SDG objectives and efficiency gains from services that have multiple impacts including and beyond health. How this is done – through integrated services or through multiple avenue provision – needs to be understood in greater depth. Recent research finds economic “development synergies” that may assist in operationalizing such multisectoral service delivery 40. Our data suggest the imperative for such combined provision as a standard of care.

It is important to note a number of study limitations. First, clinic files showed extremely low rates of viral load testing, with only a third of adolescents having any viral load record in the two years prior to recruitment. Rates of viral suppression failure among adolescents who had not been tested are unknown. This may have led to an underestimate of virological suppression failure in the present sample. Second, the viral load data was recorded in clinic files within two years prior to our data collection, which introduces problems of temporality. Third, rates of TB‐testing recorded in clinic files were also exceptionally low – consequently we used self‐reported TB diagnosis without treatment, and self‐reported symptom‐based screening, shown in Zimbabwe to have diagnostic value comparable to sputum testing among a large sample of people living with HIV. It would be of value for future studies to conduct independent TB‐testing of adolescents. These limitations reflect some of the challenges of conducting research in real‐world health services in Africa, outside high‐quality teaching hospitals and donor‐funded clinics. Fourth, provisions aligned with two of the SDGs (end poverty and end hunger) were combined due to conceptual overlap. Whilst there are plausibly separate pathways to mortality risk through food insecurity and socio‐economic status, combining these was desirable for two reasons: (i) most poverty scales (including the one applied in this study) include food security items; and (ii) the two scales were correlated (Pearson's r = 0.166, *p* < 0.0001). Fifth, the study was cross‐sectional and thus causal relationships cannot be determined, with the potential risk that some covariates may be on the causal pathway between exposure and outcome (although none of the covariates were significantly associated with the outcome in multivariate models). Future research should examine these associations using longitudinal data. Sixth, the study took place in only one country and generalizability to other countries and regions is unknown. Last, we only tested a limited selection of SDG‐aligned provisions for which we had data in this existing study, and it will be of great value for future SDG‐focused studies to examine associations of additional provisions with adolescent HIV outcomes.

The study also has notable strengths. This is the largest known study of adolescents living with HIV to include social, economic and clinical outcomes. It is the only known study that traces ART‐initiated adolescents into their communities, and in doing so includes those who have defaulted on treatment or no longer attend healthcare. Total population sampling was employed within more than 50 healthcare facilities, including hospitals and rural and urban primary health clinics – reflecting a typical range of facilities providing care to adolescents living with HIV within a resource‐limited area of South Africa.

## 5 Conclusions

We are at a point of both crisis and potential. On the most basic measure of survival, adolescents living with HIV are among the global populations “furthest behind.” Sustainable Development Goal 3 includes the ending of the epidemics of AIDS and tuberculosis (SDG 3.3) – a crucial target to ensure the health and well‐being of millions in Southern Africa. This study's findings demonstrate that this aim cannot be conceptualized within the goal of health alone 41. Instead, findings suggest that service provisions aligned with a range of SDGs are strongly associated with reduced potential mortality risk, and that combinations of protective provisions are more effective than any single factor alone. As the SDGs progress from aspirations into policies and programmes, it is essential that we develop a strong evidence‐base of SDG‐aligned services, as well as national planning and fiscal environments that support access to these services for adolescents living with HIV.

## Competing interests

The authors declare no competing interests.

## Authors’ contributions

LC, MP and ET had responsibility for the overall study design and management. LC, MP, MO and LS had responsibility for conceptualizing the paper. MO, LC and MP conducted the analyses for the paper. LC, LS, SM, MP and ET wrote the paper. All authors reviewed and approved the final version.
